# Incompatible Prescriptions

**Published:** 1887-04

**Authors:** 


### *INCOMPATIBLE PRESCRIPTIONS.*

The following examples of untoward results following the combination of familiar prescriptions are given in the *Deutsch-Amerikanische Apotheker-Zeitung* for January 15, 1887, as original with Ch. T. P. Fennel in the "Principles of Pharmacy." While these incompatibilities may be well known, yet their repetition as a reminder will not be devoid of interest.

A common and dangerous action is that resulting from the mixture of alkaloids with alkalies or alkaline salts, of which the following is an example:

℞ Strychninæ sulphat., - - - gr. ss.<br>

Elixir. brom. chloral, - - - ʒ viii. M.

Sig.—A teaspoonful morning and evening.

The result of this mixture is a colorless solution, from which is deposited in a few hours a crystalline precipitate, which is the greater portion of the strychnine ordered. Although the elixir is not officinal, it is frequently prescribed, and its potassium bromide will deposit the strychnine as an insoluble bromide. The last dose taken, unless great care to shake the mixture well was observed, would result disastrously.

The following prescription may also result badly:

℞ Morphin. sulphat., - - - gr. ii.<br>

Liq. ammon. acet., - - - - f ʒ ss.<br>

Aqu. destillat.,<br>

Syr. simpl., - - - - āā f ʒ ii. M.

Sig.—Teaspoonful doses as ordered.

When the diluted acetic acid has been neutralized, and an excess, although not great, of ammonium carbonate is present, the precipitation of the alkaloid will result as in the first instance.

The following may also prove incompatible:

℞ Potass. iodid., - - - - ʒ i.

Spts. æther. nitros.,

Aquæ, - - - - - āā ℥ i. M.

When the spiritus ætheris nitrosi is not free from nitric and acetic acids, (a result which is produced by the action of the air,) iodine will be set free, and a colored solution take the place of a colorless one.

A familiar example of decomposition occurring in the organism when chemical substances are given, is found in the simultaneous use of calomel and potassium iodide, as follows:

℞ Hydrarg. chlor. mit., - - - gr. iv.
Sacchar. lactis, - - - gr. x. M.
F. pulv. No. 10.
Sig.—A powder three times daily.

Also

℞ Potass. iodid., - - - - ℥ i.
Aqu. destillat., - - - - - ℥ iv. M.
Sig.—Teaspoonful every three hours.

A re-action between the calomel and potassium iodide occurs in the stomach, and bad results, through the irritating mercurial formed, may occur.

An explosion or unfortunate formation of gas may result from this prescription:

℞ Ammon. carbonat., - - - ℈ ii.
Syrup. scillæ, - - - - f ℥ i.
Syrup. senegæ, - - - - ℥ i. M.

The acetic acid in the syrup of squill has been overlooked in this combination; its union with the ammonium salt would result unfortunately through the liberation of carbonic acid gas.

In those cases where ingredients are ordered which even in a measure tend to produce insoluble compounds, the ingredients should be diluted as much as possible, that the resulting coagulum or compound may be easily dissipated by shaking. This is illustrated by the prescription:

| | | |
|---|---|---|
| ℞ | Liq. ferri chlorid., | ʒ iss. |
| | Muc. gum. arab., | f ℥ i. |
| | Aqu. destillat., | f ʒ iv., |

i n which the iron and mucilage should both be diluted before mixing.

The same caution obtains when tannin or liquiritia is combined with metallic or alkaloidal salts.

| | | |
|---|---|---|
| ℞ | Extr. hyoscyami aq., | ʒ ss. |
| | Tinct. valerian., | ℥ iii. |
| | Spir. ætheris nitrosi, | ʒ vi. M. |

In the above, the extract is to be diluted before mixing with the spiritus ætheris nitrosi.

The prescription,

| | | |
|---|---|---|
| ℞ | Tinct. iodin., | ʒ i., |
| | Aquæ, | ℥ i., M., |

will result in the separation of free iodine; to prevent this, potassium iodide should be added.

The prescription,

| | | |
|---|---|---|
| ℞ | Potass. chlorat., | ℈ i., |
| | Acid. hydrochlor., | ʒ ii., |
| | Aq. destillat., | ℥ x., M., |

results in different ways. If the potassium chlorate is immediately added to the acid, and then water mingled with the resulting fluid, chlorine gas is formed in solution. If the potassium chlorate is first dissolved in water, and then the acid is added, free chloric acid results.—*Therapeutic Gazette.*

